# Design of trials in lacunar stroke and cerebral small vessel disease: review and experience with the LACunar Intervention Trial 2 (LACI-2)

**DOI:** 10.1136/svn-2023-003022

**Published:** 2024-04-03

**Authors:** Gordon Blair, Jason P Appleton, Iris Mhlanga, Lisa J Woodhouse, Fergus Doubal, Philip M Bath, Joanna M Wardlaw

**Affiliations:** 1 University of Edinburgh, Edinburgh, UK; 2 Stroke Trials Unit, Division of Clinical Neuroscience, University of Nottingham, Nottingham, UK; 3 Stroke, Nottingham University Hospitals NHS Trust, Nottingham, UK

**Keywords:** Stroke, Clinical Trial, Cognitive Dysfunction, Cerebrovascular Disorders

## Abstract

Cerebral small vessel disease (cSVD) causes lacunar stroke (25% of ischaemic strokes), haemorrhage, dementia, physical frailty, or is ‘covert’, but has no specific treatment. Uncertainties about the design of clinical trials in cSVD, which patients to include or outcomes to assess, may have delayed progress. Based on experience in recent cSVD trials, we reviewed ways to facilitate future trials in patients with cSVD.

We assessed the literature and the LACunar Intervention Trial 2 (LACI-2) for data to inform choice of Participant, Intervention, Comparator, Outcome, including clinical versus intermediary endpoints, potential interventions, effect of outcome on missing data, methods to aid retention and reduce data loss. We modelled risk of missing outcomes by baseline prognostic variables in LACI-2 using binary logistic regression.

Imaging versus clinical outcomes led to larger proportions of missing data. We present reasons for and against broad versus narrow entry criteria. We identified numerous repurposable drugs with relevant modes of action to test in various cSVD subtypes. Cognitive impairment is the most common clinical outcome after lacunar ischaemic stroke but was missing more frequently than dependency, quality of life or vascular events in LACI-2. Assessing cognitive status using Diagnostic and Statistical Manual for Mental Disorders Fifth Edition can use cognitive data from multiple sources and may help reduce data losses.

Trials in patients with all cSVD subtypes are urgently needed and should use broad entry criteria and clinical outcomes and focus on ways to maximise collection of cognitive outcomes to avoid missing data.

## Introduction

 Cerebral small vessel disease (cSVD) causes a quarter of ischaemic strokes, most haemorrhagic strokes in older people, 20% of dementias and many gait, balance and mood disorders. cSVD is also common in mixed dementia pathologies and accounts overall for some 45% of dementias. cSVD can also be diagnosed on brain imaging performed for other reasons in patients with no formal clinical diagnosis, so-called ‘covert’ cSVD. Imaging features include small subcortical (‘lacunar’) infarcts, white matter hyperintensities (WMH), lacunes, microbleeds, perivascular spaces and a range of subvisible tissue changes detectable with various MRI techniques.^1 2^ The presence of cSVD features on neuroimaging, whether covert^3^ or in patients who had a stroke,^4^ increases the future risk of stroke and dementia several-fold. Thus, cSVD presents to a range of clinical services or is detected serendipitously, causes a large concurrent disease burden and substantially increases future disease risk.

Despite this, and well-known treatable risk factors (hypertension, smoking, diabetes), there are few established treatments for cSVD, of any subtype, that definitely reduce adverse clinical outcomes. Although antihypertensive treatment is essential management in patients with hypertension, it has proved difficult to show that any particular antihypertensive drug or blood pressure (BP) target reduces recurrent lacunar stroke or cognitive impairment.^5 6^ Long-term antiplatelet drugs are advised in ischaemic stroke prevention guidelines; however, few long-term secondary prevention trials reported results by stroke subtype, long-term dual antiplatelet drugs were hazardous after lacunar stroke,^7^ and antiplatelet drugs are discouraged in covert cSVD.^5^

The evidence gap reflects several factors, including limited understanding of cSVD pathology^8 9^ although endothelial dysfunction is postulated. Recently, the LACunar Intervention Trial 2 (LACI-2) showed that treatment to improve endothelial function with isosorbide mononitrate (ISMN, a nitric oxide (NO) donor), and cilostazol (a phosphodiesterase-3 (PDE3) inhibitor), for 1 year, could reduce recurrent stroke, dependency and cognitive impairment after lacunar ischaemic stroke.^10^ A treatment that improves disease outcomes is likely to be working on the underlying cause of the disease, that is, endothelial dysfunction in the case of SVD. These encouraging results can help accelerate trials in cSVD using endothelial-active drugs by repurposing other drugs, wider testing of ISMN and cilostazol in other cSVD presentations (perhaps even including haemorrhagic cSVD), or developing novel agents to improve endothelial function (table 1).

**Table 1:** Exemplar randomised controlled trials, and substudies where relevant, targeting people with cerebral small vessel disease including those with lacunar infarcts, white matter hyperintensities or sporadic intracerebral haemorrhage

Intervention	Trial	Target population	N	Outcome	FU (months)	Comment
**Pharmacotherapy**						
Anti-inflammatory, minocycline	MINERVA^69^	LACI and WMH	44	BBB permeability, microglia activation	3	Completed
Antiplatelet, dual vs mono	SPS-3^6^	LACI (on MRI)	2916	CASI	60	Neutral
BP lowering, perindopril±indapamide	PROGRESS^70^	Stroke (IS, ICH)	6105	Dementia, cognitive decline	47	Dementia/cognitive decline reduced
	PROGRESS^47 71^	Stroke (IS, ICH)	192	WMH	36	WMH progression reduced
BP lowering, telmisartan	PRoFESS^54^	Stroke (IS)	771	WMH	28	Neutral (but no BP difference)
BP lowering, intense v guideline	INFINITY^43^	WMH	199	Gait speedWMH	36	NeutralReduced
	LEOPOLD (NCT02472028)	cSVD	820	WMH	36	Ongoing
	PODCAST^72^	IS, ICH	83	ACE-R	6–30	Neutral
	PRESERVE^44^	LACI+WMH	62/111	Cerebral perfusion	3	Neutral
	PRESERVE^73^	LACI+WMH	111	White matter diffusivity, cognition	24	Neutral (SBP −6.8 mm Hg)
	PROHIBIT-ICH (ISRCTN23416732)	ICH	112	MoCA	12	Ongoing
	SPRINT-MIND^74^	Hypertension (no diabetes/stroke)	9361	Dementia, cognitive impairment	40	Neutral, but less cognitive impairment
	SPRINT-MIND^50^	Hypertension (no diabetes/stroke)	454	WMH	48	Less increase in WMH volume, and more decrease in total brain volume (but diuretic effect?)
	SPS-3^6^	LACI (on MRI)	2916	CASI	60	Neutral
GABA partial agonist (tramiprosate)	(NCT00056238)	CAA	24	Microbleeds	3	Neutral
Lipid lowering, pravastatin	PROSPER^52^	Vascular risk factors	535	New infarcts, WMH	33	Neutral
Lipid lowering, intense v guideline	PODCAST^75^	IS	77	ACE-R	6–30	Neutral. Post hoc analysis positive^75^
Neurotransmission modulators (DL-3-n-butylphthalide)	(ChiCTR-TRC-09000440)^76^	cSVD+VCI	281	ADAS-cog, CIBIC-plus	5.5	Cognition and global functioning improved
Nitric oxide donor (ISMN)	LACI-1^11 35 77^	LACI		Safety, tolerability	2	Safe, tolerable
	LACI-2^10^	LACI	363	Feasibility	12	Composite, cognition, function
PDE3-I (cilostazol)	ECLIPSE^78^	Acute LACI	130	Pulsatility index, WMH	3	Reduced pulsatility index. Neutral for WMH
	LACI-1^11 35 77^	LACI		Safety, tolerability	2	Safe, tolerable, WMH reduced
	LACI-2^10^	LACI	363	Feasibility	12	Composite, cognition, function
	Lee *et al*^79^	AD with WMH	36	WMH	6	Improved regional cerebral metabolism
PDE5-I (tadalafil)	PASTIS^80^	LACI/TIA+lacunes/WMH	55	Change in CBF	Single dose	Non-significant increase in CBF
Uric acid lowering (allopurinol)	XILO-FIST^81^	IS/TIA	464	WMH	24	Neutral, safe
**Device**						
Remote ischaemic conditioning	Liao^82^	Subcortical VaD	37	Neuropsychological profile	6	Safe but neutral
**Exercise**						
Aerobic dance	ADTSVD^83^	cSVD	110	Cognition, mood, mobility	6	Inconsistent benefits on memory and executive function
**Multi-domain**						
Nurse-led multidimensional cardiovascular intervention	preDIVA^84^	Age 70–78 years	3526	Dementia and disability, WMH	80	Neutral on all outcomes. Cluster design, 116 practices

AD, Alzheimer’s disease; ADAS-cog, Alzheimer’s Disease Assessment Scale-Cognitive Subscale; ADCS-CGIC, Alzheimer’s Disease Cooperative Study-Clinical Global Impression of Change; BBB, blood-brain barrier; BMI, body mass index; BP, blood pressure; CAA, cerebral amyloid angiopathy; CASI, Cognitive Abilities Screening Instrument; CBF, cerebral blood flow; CIBIC-plus, Clinician’s Interview-Based Impression of Change Plus caregiver input; cSVDcerebral small vessel diseaseDSST, digit symbol substitution test; FU, follow-up; ICH, intracerebral haemorrhage; IS, ischaemic stroke; LDL-c, low density lipoprotein-cholesterol; MoCAMontreal Cognitive AssessmentMRI-BOLD, MRI blood oxygenation level dependent; PDE3-I, phosphodiesterase-3 inhibition; PDE5-I, phosphodiesterase-5 inhibition; RCTrandomised controlled trialSBP, systolic BP; TIA, transient ischaemic attack; VADAS-cog, Vascular Dementia Assessment Scale-Cognitive Subscale; WMD, white matter disease; WMH, white matter hyperintensities

Here, we discuss points learned from LACI-1^11^ and LACI-2^10^ that are relevant to improving future cSVD trials design^5 12^ and accelerate finding effective treatments to improve cSVD clinical outcomes.

## Methods

We considered several practical questions on trial design and sought data to answer these from the literature, LACI-2 and other recent cSVDs trials.^10 13^ This included specific cSVD characteristics, particularly the key baseline variables to characterise the population, outcome event rates that impact on trial design, pros and cons of different clinical or imaging outcomes, ways to maximise sample size and minimise data losses, usual prescribed treatments to lacunar ischaemic stroke or cognitive cSVD patients that might interact with trial drugs, drugs that could be tested now, and ways to improve statistical efficiency.

### Comparison of imaging and clinical outcomes in trials in cSVD

We assessed the literature for randomised controlled trials (RCTs) in patients with cSVD that provided clinical and imaging endpoints to compare the use of clinical to imaging outcome measures. This included RCT’s with cSVD as an inclusion illness but also studies that used a specific SVD outcome (eg, trials of patients with cardiovascular risk factors that also assessed cSVD as an outcome). Trials broadly fell into three categories: RCT’s of participants with cSVD with clinical outcomes only; RCT’s using a cSVD imaging marker as a main outcome; and RCT’s with a main trial using clinical outcomes with imaging substudies assessing an cSVD imaging marker. We compared the number of participants randomised, number of centres and the follow-up rate in these studies.

To identify studies, we reviewed trials listed in the recent ESO guidelines on covert cSVD,^5^ which provides a comprehensive review of relevant RCT’s in cSVD. We reviewed all the included references to identify RCT’s that provide exemplar data on the differences between trials using clinical and imaging outcomes. We also searched MEDLINE and EMBASE combining a search of cSVD terms with a search for RCTs to identify any further studies that may be relevant but were not included in the guideline. Terms included: ‘lacunar stroke’ OR ‘lacunar ischaemic stroke’ OR ‘small vessel disease’ OR ‘cerebral small vessel disease’ OR ‘stroke’ OR ‘SVD’ OR ‘white matter hyperintensity’ OR ‘white matter lesion’ OR ‘lacune’ OR ‘memory’ OR ‘cognition/ve’ OR ‘dementia’ AND ‘randomised clinical trial’ OR ‘clinical trial’ AND ‘magnetic resonance imaging’ OR ‘MRI’ OR ‘CT brain scan’ OR ‘computed tomography’.

### Assessment of outcomes using remote methods

To identify alternative methods of performing follow-up that might improve trial compliance, drug adherence and reduce missing data, we searched PubMed, review papers and guidelines for trials that tested remote technology-based outcomes, using the above terms for cSVD and the following terms for remote devices: AND ‘remote’ OR ‘remote technology’ OR ‘wearable technology’ OR ‘device’ OR ‘monitor’ OR ‘watch’ OR ‘tablet’ OR ‘telephone’ OR ‘mobile’ OR ‘mobile application’ OR ‘mobile app’ OR ‘video’.

### Follow-up in LACI-2, representing a cSVD trial in lacunar stroke

We analysed data from the LACI-2 RCT (full trial methods including regulatory approvals see Wardlaw *et al*^10^) to assess follow-up rates for the clinical outcomes used in the trial and factors associated with missingness. LACI-2 assessed cognitive function by mapping cognitive test data to the Diagnostic and Statistical Manual for Mental Disorders Fifth Edition (DSM-5) ordinal scale of neurocognitive disorders. This approach attempted to minimise data loss by avoiding the problem that cognitive test data (eg, Montreal Cognitive Assessment (MoCA)) are most likely to be missing in those with more severe disease, for example, stroke. We therefore assessed which outcomes were most likely to be missing at follow-up and how this impacted on whether a cognitive outcome could, or not, be derived. We also used binary logistic regression models, on derived missing versus no missing outcome variables, to assess which factors at baseline (age, sex, prestroke modified Rankin Scale (mRS), National Institutes of Health Stroke Scale (NIHSS), systolic BP, smoking status, time to randomisation and education level) might predict the data that are most likely to be missing at final follow-up. We used data from all 363 randomised participants, noting that 5 participants were lost or withdrew thus providing data on 358 participants at 12 months.

## Results

### General considerations when designing trials in cSVD (panel 1)

cSVD develops slowly and is a long-term condition. Therefore, any intervention for primary or secondary prevention will likely have to be given long-term. Since cSVD is common with a high societal burden, interventions should be of modest cost to be affordable. For practical reasons, interventions will need to be oral, transdermal or nasal, preferably with daily (or less) administration.^14^

Patients with cSVD presentations typically have several vascular risk factors for which they may be on several drugs in addition to drugs for other age-related conditions (arthritis, gastrointestinal disorders), making polypharmacy common in cSVD patients,^15^ and it important to consider common drug interactions in trial design and to increase the likelihood of adoption into clinical practice if the intervention is effective.^16^

Comorbidities increase with age and socioeconomic disadvantage and thus are common in cSVD. Restrictive inclusion criteria may result in many patients being excluded through having comorbidities, in turn restricting the trials’ generalisability. A large meta-analysis did not find evidence that comorbidities modify treatment effect,^17^ so there is no reason to exclude patients on the basis of comorbidities alone.^18^

cSVD can present in many ways and mimic other disorders, while both clinical and brain imaging diagnosis are imperfect.^2^ A clinical lacunar stroke syndrome may be mistaken for a cortical ischaemic stroke syndrome and vice versa,^19^ especially when acute.^20^ In patients with an acute clinical lacunar stroke syndrome, brain imaging may show a recent small subcortical infarct in a brain region relevant for the symptoms, but even sensitive diffusion-weighted imaging MRI will not show a definite recent small subcortical infarct in up to 30% of patients for several reasons.^21^

Most small subcortical infarcts are due to intrinsic small vessel disease; about 15%–20% can result from emboli from proximal atheroma or the heart^22^ or intracranial artery stenosis/occlusion (‘branch atheromatous disease’).^23^ While investigations can help exclude athero-embolic and cardio-embolic causes, common pathologies may coexist. It can be impossible to tell ultimately if a particular small subcortical infarct was due to intrinsic disease or not. Similar problems occur with cSVD presenting to cognitive clinics where overlap of vascular and other neurodegenerative causes of cognitive impairment is common. Therefore, the level of specificity of diagnosis is a key feature of the cSVD trial design since it will affect the amount of screening or additional investigation required to identify patients, number of exclusions, trial costs, duration and generalisability back into clinical practice.

The long-term nature of cSVD requires long trial duration making retention a key issue. Potential ways to maintain retention include minimising burden on participants by reducing travel and inconvenience, using telephone/video follow-up rather than face-to-face requiring trips to hospital or home visits by researchers (either way, travel adds cost and time), ensuring follow-up visits are as short as practicable and minimising imaging follow-up. Adherence can be encouraged through education about study drug side-effects, escalating doses of drugs gradually and offering dose reduction to a tolerable level to manage side-effects, as used in LACI-1 and LACI-2.^11 24^

**Figure 1:**
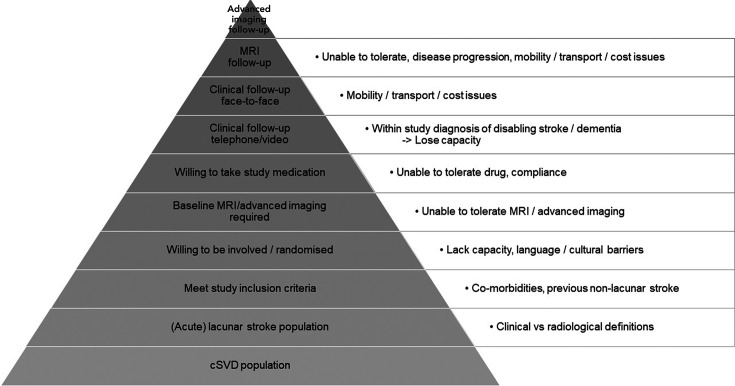
The ‘inverted pyramid of perfection’ in trial recruitment and follow-up: effect of increasing levels of selection and follow-up methods on participant numbers and generalisability. cSVD, cerebral small vessel disease.

Several potential factors influencing screening, recruitment, retention, feasibility, delivery and ultimately the generalisability of cSVD trials, are highlighted in figure 1.

### Information needed to plan a trial in cSVD

The following Participants, Intervention, Comparator, Outcome points need to be considered carefully when planning a trial in patients with cSVD.^12^

#### Participants

The clinical presentation (stroke, cognitive, mobility, mood, covert, all of these) will influence which clinics (or wards) to recruit from, and how to define the population of interest. It will affect: usual prescribed medications, and whether these might interact with the trial drug; likely adherence to trial drug, whether participants have capacity to consent or are dependent which may affect compliance with trial procedures; whether they are at risk of losing capacity during the trial and, if so, how this will be managed.

Defining the population will variously include participants based on clinical diagnosis, for example, lacunar stroke syndrome^25^ backed by imaging to confirm a small subcortical infarct and/or exclude other causes, as in LACI-1 and LACI-2.^10^ Mechanistic classifications (eg, TOAST), though widely used, require more investigations, and may leave a significant proportion of patients unclassified, delay recruitment, restrict the trial to highly specialist centres, and impede generalisablity.^26^ The definition will, in part, determine which baseline assessments are needed, whether these are part of routine clinical assessment or specific for the trial. The more trial-specific assessments, the more burdensome the trial for sites, patients and funders.

Generalisability from the ‘trial population’ to ‘most patients with the disease of interest’ is key. Hence, perfection should not impede delivery: comorbidities are common in cSVD but there is no evidence that they modify treatment effect,^17^ so there is no reason to fall prey to the dwindling recruitment seen in the ‘inverted pyramid of perfection’ (figure 1). Excessive exclusion and tight inclusion criteria result in an ungeneralisable study population. The need for MRI or other advanced imaging may restrict recruitment, for example, due to intolerance, and add delays: their use should be fully justified.

Minimisation at randomisation is recommended to reduce imbalances in baseline characteristics between randomised groups^27^ and adds statistical power.^28^ LACI-2 used age, sex, NIHSS, mRS, time since stroke, educational attainment, BP and smoking status, each are key outcome predictor in cSVD.^29 30^ Minimisation variables should also be used as covariates during analysis; we added baseline MoCA when analysing cognitive outcomes in LACI-2.^24^ If feasible, minimisation should also include an estimate of cSVD lesion severity (eg, WMH score, presence of lacunes or microbleeds) as appropriate for site experience^24 31^ since rapid central adjudication is likely to delay recruitment and increase complexity.

#### Intervention and comparator

Table 1 lists trials in patients with cSVD including the intervention(s) tested, illustrating many potential repurposable agents that are well primed for testing in future trials.^14^ Assessing repurposed agents is generally easier than novel agents due to the former’s known safety profile and interactions with prescribed guideline drugs.

Some interventions can no longer be recommended for testing, including clopidogrel-based dual antiplatelet therapy where harm was identified.^6^ Intensive BP-lowering has been assessed in multiple studies with variable results (reviewed in Wardlaw *et al*^5^). One interpretation is that while lowering BP is effective in reducing cognitive decline in mixed populations of ischaemic and/or haemorrhagic stroke, it is not effective in pure populations with cSVD (table 1), this assuming that sample sizes are sufficient (typically several thousands of participants) and that adequate BP lowering is achieved and for a sufficient time (typically 3 or more years).

Although other drug interventions have not been studied in large trials or for long periods of time, these provide useful guidance as to the type of interventions that might show promise, including anti-inflammatory agents, neurotransmission modulators, NO donors and phosphodiesterase inhibitors (table 1). We have previously reviewed other mechanisms that may be relevant in moderating cSVD.^14^

Whether or not to continue usual guideline treatments during the trial requires consideration. LACI-2 started planning in 2015 and recruiting in 2018 when we could not justify withholding guideline secondary stroke prevention from patients with clinically evident lacunar ischaemic stroke: patients with lacunar ischaemic stroke had been grouped with other ischaemic stroke subtypes in stroke prevention trials, with no specific guidelines or other good evidence to the contrary. Secondary stroke prevention in ischaemic stroke includes an antiplatelet drug (usually clopidogrel or aspirin), antihypertensive and lipid lowering therapy. Patients with atrial fibrillation (which is unusual in lacunar ischaemic stroke, but common disorders can co-occur) usually take an anticoagulant (usually direct oral, eg, apixaban). Therefore, in LACI-1 and LACI-2, all patients continued their prescribed guideline-based stroke prevention, which made the comparator ‘best guideline-based medical therapy’. To avoid the issue of anticoagulant interactions with cilostazol, a mild antiplatelet, patients on anticoagulants could be randomised to just ISMN. More thought will be needed if a novel drug is being tested where knowledge on adverse events and drug interactions is lacking.

Adherence to long-term prescribed medication is a major problem, for example, the WHO data indicate only 50% of patients with chronic diseases adhere to treatment recommendations, consistent with a systematic review of 69 137 patients in 29 studies where non-adherence to secondary prevention was 30.9% (95% CI 26.8% to 35.5%), with trends to associations with disability (OR 1.27, 95% CI 0.93 to 1.72), polypharmacy (OR 1.29, 95% CI 0.9 to 1.9) and age (OR 1.04, 95% CI 0.96 to 1.14).^32^ Strategies such as incrementing the dose gradually and allowing patients to take a lower than full trial drug dose, may help reduce side effects and be appropriate. This approach, tested in LACI-1,^11^ worked well in LACI-2, where most participants remained on over 75% of the full dose of trial drug for 1 year.^10^

A flexible approach, aiming to include rather than exclude patients, may help maximise recruitment and generalisability and avoid issues due to polypharmacy,^15^ for example, allow randomisation to just one arm of the trial to avoid contra-indications, or allow a lower dose, to mirror real life.

Guideline ‘drift’ may be a problem, for example, if a trial drug becomes recommended in the course of the trial changing the ‘usual prevention treatment’, it will become impossible to continue randomising to that drug and may alter the comparator,^33^ or affect potential interactions with the trial drug. Choice of the trial drug and control should consider whether a change in usual prescribed medications is likely during the course of the trial so that the impact of any change can be minimised via the trial design.

#### Outcome(s)

The expected outcome event rates or distributions affect sample size,^34^ power and cost. The clinical outcomes of concern in most cSVD subtypes are: (1) stroke, recurrent or first, ischaemic or haemorrhagic; (2) cognitive decline or dementia; (3) dependency; (4) death; (5) major adverse cardiovascular events; (6) mobility problems including poor balance; (7) mood disorders including depression; and (8) adverse events such as haemorrhage, falls, and dizziness.

Several key questions arise: first, how to collect the primary outcome, for example, at clinic or remotely to reduce cost and potentially increase compliance. Second, timing, which will influence duration of follow-up and cost. Third, whether the outcome is sensitive to therapeutic change. Fourth, is a relative or caregiver needed to get the outcome?

**Figure 2:**
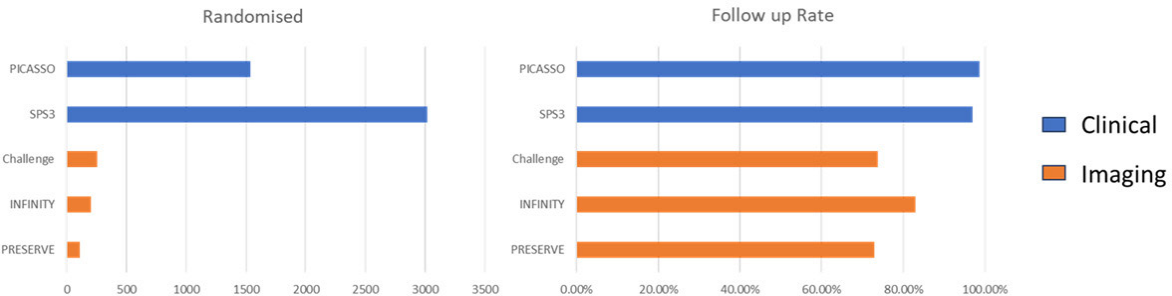
Number of participants randomised and follow-up rate achieved in example randomised controlled trial’s that used clinical or imaging outcomes.

Intermediary outcomes from imaging, for example, WMH change, total cSVD lesion change, diffusion tensor imaging (DTI) measures, blood markers or other physiological measures, are popular in phase 2 trials and may give early proof of concept.^35^ However, they should not replace clinical outcomes in phase 3 trials: they are less important to patients, may lack standardisation (figure 2), may reduce patient participation, restrict sites that can participate (table 2), suffer from data losses, inflate trial cost, increase data processing, bias the trial outcome and restrict generalisability. Most importantly, they may not reduce sample size^12^ since signal-to-noise may not increase and some markers, for example, WMH, can increase or decrease.^36^ Careful thought is required as to when an intermediary marker will be truly useful and justified.

**Table 2:** Trials with clinical and imaging outcomes: comparing numbers screened, randomised, centres, recruitment rates, completeness of follow-up

	PROGRESS		VITATOPS		SPRINT/SPRINT MIND		PROSPER		PROFESS		ACCORD/ACCORD MIND		LACI-1	
	Clinical	Imaging	Clinical	Imaging	Clinical	Imaging	Clinical	Imaging	Clinical	Imaging	Clinical	Imaging	Clinical	Imaging
Screened	7121	323	N/A	N/A	14 692	1267	23 770	1100	N/A	N/A	19 716	N/A	N/A	N/A
Randomised	6105	254	8164	471	9361	673	5804	646	20 333	1057	10 251	632	57	27
Follow-up complete	6102 (99.9%)	192 (75.6%)	7462 (91.4%)	359(76.2%)	8563 (91.5%)	454 (67.5%)	5147 (88.7%)	554 (85.8%)	20 208 (99.4%)	771 (72.9%)	10 201 (99.5%)	503 (79.6%)	56 (98.3%)	22 (81.5%)
Centres	172	10	123	5	101	7	3	1	695	?	77	28	2	1
Recruitment rate	1.18	0.85	0.55	3.77	3.31	3.43	113.8	38	0.86	?	3.69	0.81	1.67	1.59
Follow-up duration	46.8	36	40.8	25	39.1	6.5	38.4	33	30	27.9	42	35	3	3
Age (mean)	64	60.8	62.6	64.3	67.9	67.3	75.4	75	66.2	65.4	62.2	62.4	66.1	68

Trial outcomes should focus on those that are of most concern to patients. In cSVD, patients repeatedly list cognitive decline as their primary concern, with lost independence and recurrent stroke following in importance. This reflects that cognitive impairment is the most common outcome after lacunar ischaemic stroke,^37^ as in LACI-2.^10^ Cognitive decline is also a major concern to patients with other types of cSVD and stroke.^38^

### Improving efficiencies in cSVD trials: outcomes, missingness, adherence and analysis

A large sample size provides the most generalisable results and ‘trumps’ most other design considerations^39^; the larger the sample, the more likely that the trial will provide a definitive result, be able to examine important prespecified subgroups and be generalisable to clinic populations. There is no point in having a very sensitive or specific outcome that can only be assessed in a small proportion of the population, or if collecting it discourages people from participating in the trial, or of ever having the treatment. Alongside methods to simplify and optimise recruitment, it is important to consider how to minimise data losses in the population of interest. Here we consider data losses, drug adherence and statistical approaches to improve trial efficiency.

#### Clinical or imaging outcomes?

The Secondary Prevention of Small Subcortical Stroke trial^40^ (patients with clinically evident lacunar ischaemic stroke) and the PICASSO^41^ trial (ischaemic stroke with microbleeds) used clinical outcomes and recruited 3020 and 1534 participants, respectively with follow-up rates of 98.6% and 97%. The CHALLENGE^42^ (radiological evidence of cSVD), INFINITY^43^ (age over 75, hypertensive and WMH on MRI) and PRESERVE^44^ (lacunar stroke, hypertension and confluent WMH) used imaging outcomes as primary endpoints and recruited 256, 199 and 111 participants, respectively, with follow-up rates of 73.8%, 82.9% and 73%.

**Figure 3:**
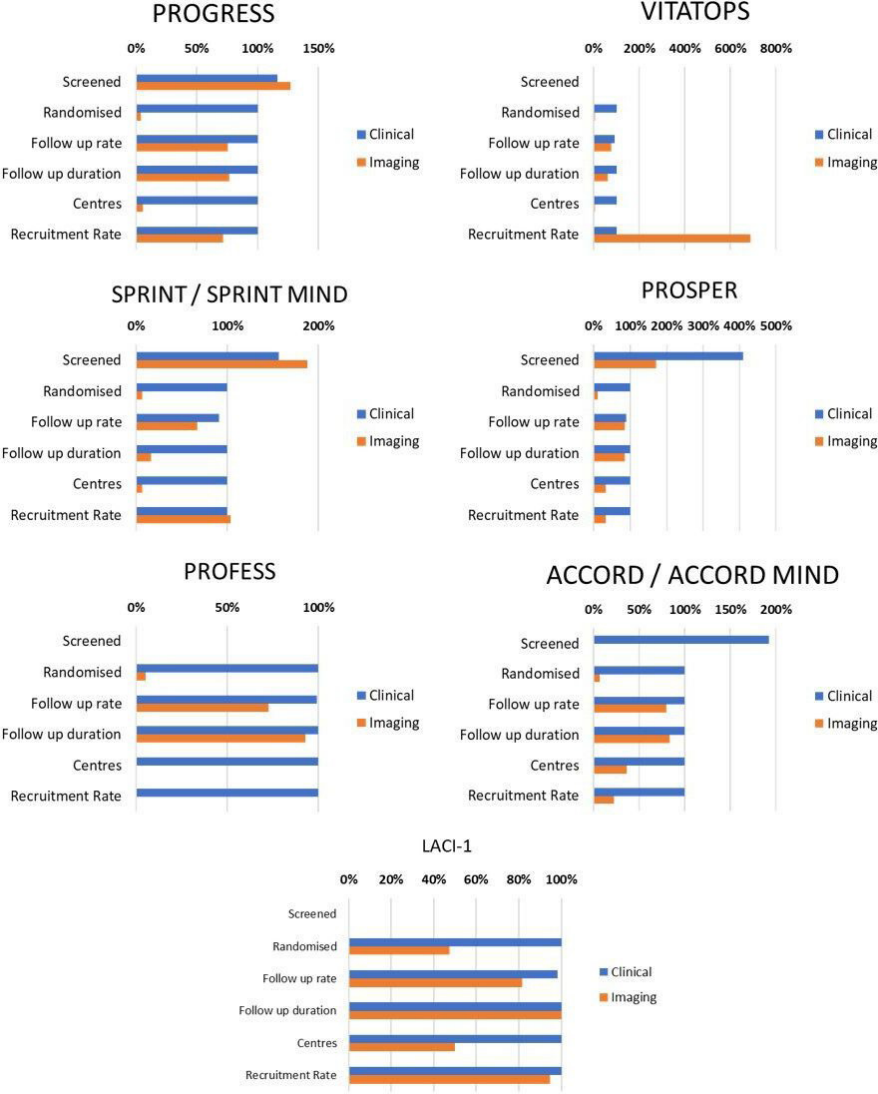
Comparison of clinical and imaging outcomes in trials with an imaging substudy.

We identified seven RCT’s with nested imaging substudies to compare studies using clinical and imaging outcomes (table 2, figure 3). Imaging substudies included between 5.2% and 47.4% of the participants in these RCTs. Completeness of data at follow-up in the clinical outcome RCTs was 88.7%–99.95%, whereas completeness of data at follow-up in the imaging substudies was 67.5%–85.8%. Additionally, follow-up duration in the imaging substudies was shorter than the clinical outcomes (16.6%–93% of the duration achieved in the clinical RCT).^1145^,^57^

Table 2, figures2  3 show that RCT’s in cSVD using clinical outcomes randomise more participants, can be done in more centres and have higher follow-up rates. Studies with imaging outcomes have much smaller sample sizes, are limited to few expert centres and have much lower follow-up rates compared with studies using clinical outcomes.

#### Minimising data losses

Losses impair trial efficiency, introduce bias and should be avoided where possible. Key factors that increase data loss (including incomplete case report forms and poor quality data) include the number of visits and the quantity of data collected at each visit, these resulting in patient fatigue and leading to drop-outs. Most studies collect far more data than they ever fully analyse or publish.

It is key to choose easy-to-collect variables, avoid long questionnaires and use proven outcomes that work in the population of interest. In large trials, outcomes that can be collected remotely, for example, by post, phone or web, or can be obtained from more than one source (eg, from a carer if the patient is unable to respond) are ideal. The primary and key secondary outcomes should be asked first in case participants tire and cannot answer later questions.

Table 3 shows the proportion of missing data for clinical outcome variables in LACI-2. 363 participants were randomised and five were lost or withdrew, leaving 358 with data at 12 months. Table 3 also shows which baseline prognostic variables most predicted missingness, according to the applied binary logistic regression models. Tests such as Trail Making were missing for many participants due to the need for in-person assessment which was disrupted by COVID-19 regulations. Many participants were reluctant or unable to provide a named informant at recruitment, limiting IQCODE data. Only 11% of data were missing for the mRS, place of residence and quality of life, while individual cognitive tests obtained by phone (t-MoCA, TICS-m, verbal fluency) and the DSM-5 7-level and 4-level neurocognitive categorisation were missing for around 15% of participants. Data on death, clinical dementia diagnosis, recurrent stroke and myocardial infarction (MI) were available for all 358 participants.

**Table 3:** Proportion and prediction of missing data for clinical outcomes assessed at 12 months in the LACI-2 trial based on key baseline variables

Outcome	Participants where outcome is missing (%)	Baseline predictors as covariates in models to predict missingness						
		Age	BaselineMoCA	Sex	Prestroke mRS	NIHSS	Time to randomisation	Education
Cog 7 Level	55 (15.15%)	*−0.038(0.018)	−0.185 (<0.0001)	0.721 (0.064)	*Overall (0.006)	−0.258 (0.057)	0.0002 (0.208)	Overall(0.257)
Cog 4 Level	55 (15.15%)	*−0.040(0.018)	−0.185 (<0.0001)	0.721(0.064)	*Overall (0.006)	−0.258 (0.057)	0.0002 (0.208)	Overall(0.257)
Modified Rankin Scale	40 (11.02%)	*−0.036 (0.045)	−0.010 (0.849)	0.021 (0.958)	Overall (0.083)	−0.187 (0.008)	−0.001 (0.272)	*Overall (0.015)
t-MoCA	56 (15.43%)	*0.036 (0.023)	*0.187 (<0.0001)	−0.585 (0.123)	*Overall (0.017)	0.188 (0.145)	−0.0004 0.168)	Overall (0.364)
TICS-m	50 (13.77%)	*0.041 (0.012)	*0.205 (<0.0001)	−0.547 (0.166)	*Overall (0.036)	0.343 (0.018)	0.0002 (0.669)	Overall (0.536)
Verbal fluency test	44 (12.12%)	−0.033 (0.056)	−0.189 (0.0001)	0.495 (0.226)	Overall (0.051)	*−0.313 (0.036)	−0.0001 (0.762)	Overall (0.620)
Trail Making Test B†	207 (57.02%)	0.003 (0.821)	0.0001 (0.999)	−0.099 (0.684)	Overall (0.164)	0.137 (0.136)	0.0002 (0.506)	Overall (0.146)
Zung depression scale	46 (12.67%)	*−0.034(0.040)	−0.063 (0.206)	−0.017 (0.963)	Overall (0.087)	−0.190 (0.159)	−0.0004 (0.378)	Overall (0.092)
Care home placement‡	42 (11.57%)	*−0.043 (0.013)	−0.035 (0.506)	0.098 (0.802)	Overall (0.067)	−0.175 (0.212)	−0.0006 (0.250)	*Overall(0.031)
IQCODE§	205 (56.47%)	*−0.023 (0.041)	0.002 (0.958)	0.115 (0.634)	Overall (0.116)	0.097 (0.280)	−0.0006 (0.019)	Overall (0.693)
EQ-5D-5L¶	43 (11.85%)	*−0.045 (0.010)	−0.055 (0.289)	−0.005 (0.990)	Overall (0.093)	−0.166 (0.227)	−0.0004 (0.380)	*Overall (0.034)
EQVAS¶	43 (11.85%)	*−0.045(0.010)	−0.055 (0.289)	−0.005 (0.990)	Overall(0.093)	−0.166 (0.227)	−0.0004 (0.380)	*Overall (0.034)

Each row corresponds to a model using column variables as predictors.

Systolic BP and smoking did not predict missing variables (data not shown).

* estimate (p value)*=overall significant predictor variable.

† : rRequired in-person assessment; many participants were unable to attend for assessment due to COVID-19 regulations.

‡ : Disposition that is, place of residence, at 12 months A months. A: home independent— 267, H: home with carer-—49, R: residential home-—1, Ddied-—4, Mmissing and Nnull- —11+35=46–4=42.

§ : IQCODE: information collected from a person who knows the participant well; 213 participants did not provide informant details at randomisation, 7 participants had IQCODE but no informant recorded, 1 participant had informant but IQCODE not computed due to missing values.

¶ : EQ-5D-5L and EQVAS— – measures of quality of life.

DSM-5Diagnostic and Statistical Manual of Mental Disorders Fifth EditionNIHSSNational Institutes of Health Stroke Scale

Increasing age affected missingness for most outcomes; baseline MoCA and prestroke mRS affected missingness of cognitive and mRS outcomes. In contrast, NIHSS, sex, time to randomisation, education, smoking and BP did not affect missingness.

The self-reported (post or phone) Stroke Impact Scale elements were missing in 12%–14% for all subdomains apart from ‘role in society’ which was missing in 21%. The DSM-5 7-level and 4-level cognition only used the t-MoCA and TICS-m specific cognitive tests; further analysis is ongoing to determine if missingness can be reduced further by using additional cognitive data (eg, verbal fluency). This highlights that cognition is difficult to collect in patients who had a stroke, even mild stroke such as lacunar stroke, and that better methods are still needed to reduce incomplete cognitive data. Web-based methods might now be more feasible for some participants than they were when LACI-2 started.

#### Could technologies help data completeness in cSVD trials?

Remote technologies could help trials in patients with cSVD and reduce costs. Digital remote capture of clinical outcomes including cognition, physical activity and apathy^58^ show promise but is unlikely to be universally applicable or acceptable, particularly to those with existing physical or cognitive disabilities. The use of remote and wearable technology to monitor physiological parameters is gaining popularity. Its application in trials in cSVD, stroke and dementia could be beneficial but requires further assessment since studies to date have been small and not specific to cSVD. A study in 73 patients with either chronic obstructive pulmonary disease (n=35) or previous stroke (n=38, mean age 59 years) who owned a smartphone, had internet access and were willing to wear a FitBit, assessed physical, cognitive and psychosocial function monthly over 3 months. Reminders were sent to participants to synchronise their devices and complete online assessments, with 65% of participants requiring at least one reminder to achieve a wear time of 77%.^59^ This small study included a selective and relatively young population, questioning the feasibility of remote monitoring in older or less healthy individuals. A recent study of 82 people (mean age 80 years) with dementia, including vascular dementia, demonstrated that remote monitoring of physiological parameters was feasible with carer support but needs confirmation.^60^

#### Medication adherence

Medication adherence is a challenge for many stroke survivors and in clinical trials. Two systematic reviews of interventions to improve adherence found 33^61^ and 182^16^ trials, respectively in which all interventions were complex involving combinations of more convenient care, information, counselling, reminders, self-monitoring, reinforcement, family therapy and other forms of additional supervision or attention. The intensity is reflected in some trials’ use of tailored ongoing support from allied health professionals (eg, pharmacists), who often delivered intense education, counselling (including motivational interviewing or cognitive behavioural therapy by professionals) or daily treatment support (or both), and sometimes additional support from family or peers. In the 2014 review, only five RCTs reported improvements in both adherence and clinical outcomes, and no common intervention characteristics were apparent.^16^ In both reviews, even the most effective of these interventions had modest effects and did not lead to large improvements in adherence or clinical outcomes.^61^ A systematic review of mobile phone intervention (four trials, 2429 patients) showed text-messaging increased adherence and reduced BP by 2–7 mm Hg.^62^ A systematic review and meta-analysis of 10 RCTs demonstrated that mobile applications and telephone reminders improved medication adherence in 2151 patients following stroke compared with usual care: applications and messaging interventions were more effective than telephone calls.^63^

#### Remote cognitive assessments by telephone or video

Remote cognitive assessments by telephone or video are increasingly used in clinical trials, although a Cochrane review found the evidence on test accuracy for diagnosing dementia was limited,^64^ supporting the use of multiple overlapping approaches. There are validated remote ‘in-person’ cognitive tests for use on smartphone or tablet devices,^65 66^ but they have not yet been applied in trials of cognitively impaired individuals. Completion of relevant follow-up questionnaires, which could also be sent to participants’ carers/relatives, could be performed online or via an app with support from a caregiver. Given that the greatest cost involved with clinical trials is staffing, if practical, such approaches could reduce the need for face-to-face follow-up visits, reserving telephone follow-up for those unable to comply with, or tolerate, remote technology.

#### Optimise statistical efficiency

Space precludes a detailed consideration of statistical efficiencies, but we mention a few principles here.

Adjust analyses wherever possible for minimisation variables^67^: this reduces imbalances between treatment groups, increases power^28^ and the likelihood of detecting true treatment effects.

Ordinal scales (eg, mRS) analysed using ordinal shift methods are more efficient than binary analysis.^68^ Most acute stroke trials now use ordinal analysis of the mRS rather than the binary ‘alive and independent versus dependent or dead’ as was common previously. Since ordinal shift analysis is more efficient, treatment effects can be detected with fewer participants for the same statistical power. Cognition may be difficult to assess after stroke due to a participant’s inability to complete elements of some tests. LACI-2 mapped individual t-MoCA and TICS-m cognitive test results, other evidence of dementia (clinical diagnosis, prescribed medication) to the DSM-5 neurocognitive disorders scale, creating a 4-level or 7-level ordinal scale which could be analysed using ordinal shift methods. This ordinal analysis (and minimising data loss) identified a potential benefit of ISMN and of ISMN+cilostazol more conclusively than using t-MoCA alone.^10^

Other approaches to increase statistical efficiency include ‘global’ analyses including all relevant outcomes (eg, the Wei-Lachin test) which increases sensitivity to detect treatment effects, but while powerful, cannot be adjusted for covariates. Alternatively, composite outcomes, for example, LACI-2 used any of stroke, MI, death, dependency, cognitive impairment to help increase the outcome event rates to gain power,^24^ but since composite analysis can only include patients with complete variables for outcomes in the composite, it paradoxically reduces the available data and power (table 3).

## Discussion

LACI-2 demonstrates that moderate-sized trials in patients with cSVD that use clinical outcomes can provide evidence of treatment benefit.^10^ LACI-2 also confirmed that cognitive impairment is the most common adverse outcome after lacunar ischaemic stroke, several times more frequent than recurrent stroke, death or dependency. Ordinal analysis of conventional cognitive tests mapped to the DSM-5 neurocognitive scale was an efficient, clinically relevant method to assess cognitive status that helped reduce impact of missing/incomplete individual cognitive tests. While further testing is needed, in principle alternative tests could be mapped to DSM-5. Future trials in patients with cSVD should consider focusing on cognitive outcomes, with dependency, recurrent vascular events, death and quality of life as secondary outcomes. Current clinical guidelines for cSVD are hampered by many small trials with imaging outcomes and insufficient large trials with clinical outcomes.^5^ Imaging outcomes are of limited relevance to patients, risk data losses, bias, may not reduce sample sizes, and no matter how sophisticated, they will not identify a positive result if the intervention is ineffective. While this review concerns cSVD, similar points may apply more generally to trials in stroke or dementia.

Trials are expensive and staff are usually the most expensive item. The longer the trial, the higher the cost, the harder it is to sustain interest and motivation, the more likely that patients will drop out, or that funders will cut the money, that staff will change risking loss of continuity, data losses, need for more training, and more inefficiencies. The more streamlined the trial design, the faster it will recruit, the faster it will be finished, and the results can benefit humanity. The analysis of published trials with clinical and/or imaging outcomes shows that with imaging outcomes, fewer centres can participate, recruitment rates are lower, sample sizes are much lower, reducing the chance of reliably detecting true effects, and missing data were more frequent (table 2, figures2  3). While imaging studies may provide insights into disease mechanisms, or evidence of direct effects of an intervention on cSVD lesions, clinical outcomes will be more applicable at scale and more generalisable. Trials using imaging outcomes should always report clinical outcomes which can then contribute to meta-analyses. Previous papers have assessed the impact of clinical or imaging outcomes on predicted sample sizes for trials in SVD.^12 36^ However, we are not aware of papers that have assessed how use of clinical or imaging outcomes may affect the practicality of achieving large sample sizes and the generalisability of results.

Other important points in LACI-2 were broad inclusion criteria, minimising randomisation on key prognostic variables, and allowing CT or MRI to diagnose the lacunar stroke which did not dilute the trial with non-lacunar stroke. The ‘inverted pyramid of perfection’ (figure 1) demonstrates the rapidly dwindling sample size caused by increasingly narrow selection criteria and adverse effect on generalisability. Minimising randomisation on baseline prognostic variables helps to ensure that treatment groups are well-balanced.^10^ In LACI-1, insisting on MRI at baseline would have prolonged recruitment by a third and cost several £100k more than by allowing recruitment of patients with CT, even in the two expert centres with good MRI access that participated in LACI-1.^11^ LACI-2 confirmed that the ‘randomise with CT’ strategy worked, since very few non-lacunar strokes were recruited, most participants had an MRI anyway and only a very tiny number did not have lacunar stroke.^10^

Broad inclusion criteria are helpful in terms of improving recruitment, but certain factors could potentially also adversely impact on outcome collection, as shown in LACI-2 (table 3). Information on predictors for missing data, if available, should therefore be reviewed when developing inclusion and exclusion criteria to minimise their impact. Furthermore, use of the factors identified in the minimisation algorithm could also ensure that the potential for missing outcome data due to pre-randomisation status is distributed equally across the groups.

Simple outcomes, that can be obtained remotely and are not reliant on obtaining the information only from the participant (eg, mRS, clinical diagnosis of dementia, activities of daily living, can be obtained from patient, relative or even routine clinical data) are more likely to be available at follow-up. Tests that require in person follow-up (eg, Trail Making Test) are very susceptible to missing follow-up data (table 3). Future trials in cSVD may consider incorporating digital follow-up methods, using mobile and/or wearable technology to improve participant retention, drug adherence, capture relevant clinical outcomes (including mobility, frailty, function and cognition using tablets/apps) and aid follow-up through digital prompts and reminders to reduce data losses. Furthermore, digital approaches could include multiple language options to broaden inclusivity. These methods may reduce the staff costs of future trials by reducing face-to-face visits and telephone follow-ups. We considered using web-based or email follow-up in LACI-2 but surveys indicated that many people did not use or have good access to relevant devices at that time. The COVID-19 pandemic increased familiarity with digital devices across the population and such methods might be more practical now and warrant further investigation to ensure such approaches do not result in further selection bias.

Translation of trial findings to clinical practice is critical, underscoring the need for trials to be as clinically relevant as possible, with meaningful outcomes for patients, that centre on future health needs. Implementation into guidelines is facilitated when trials have broad entry criteria, balance randomisation on key prognostic variables, collect clinically meaningful outcomes, and reflect the multidisciplinarity of services that typically encounter patients with cSVD.

In conclusion, large pragmatic trials in patients with cSVD and clinical outcomes are feasible, there are many relevant interventions to test (table 1), entry criteria should be broad to aid generalisability, use of remote assessments and efficient statistical methods should help increase power, reduce sample sizes and accelerate identification of effective treatments to prevent long-term adverse outcomes of cSVD. Trials might use a parallel group or platform multi-arm multi-stage master protocol approach.
